# Adaptive Properties of the Genetically Encoded Amino Acid Alphabet Are Inherited from Its Subsets

**DOI:** 10.1038/s41598-019-47574-x

**Published:** 2019-08-28

**Authors:** Melissa Ilardo, Rudrarup Bose, Markus Meringer, Bakhtiyor Rasulev, Natalie Grefenstette, James Stephenson, Stephen Freeland, Richard J. Gillams, Christopher J. Butch, H. James Cleaves

**Affiliations:** 1 University of Utah Hematology, UC Berkeley Integrative Biology, George and Dolores Eccles Institute of Human Genetics, 15 N 2030 E, Room: 3240, Salt Lake City, UT 84112 USA; 2 National Institute of Science Education and Research Bhubaneswar, P.O. Jatni, Khurda, 752050 Odisha India; 3 German Aerospace Center (DLR), Earth Observation Center (EOC), Münchner Straße 20, 82234 Oberpfaffenhofen-Wessling, Germany; 4 Department of Coatings and Polymeric Materials, North Dakota State University, Fargo, ND 58108 USA; 5 Department of Chemistry, University College London, 20 Gordon street, London, WC1H 0AJ UK; 6 European Molecular Biology Laboratory–European Bioinformatics Institute, Wellcome Trust Genome Campus, Hinxton, UK; 7 Wellcome Trust Sanger Institute, Wellcome Trust Genome Campus, Hinxton, UK; 8 University of Maryland, Baltimore County, 1000 Hilltop Circle, Baltimore, MD 21250 USA; 9 Earth-Life Science Institute, Tokyo Institute of Technology, 2-12-1-IE-1 Ookayama, Meguro-ku, Tokyo, 152-8550 Japan; 10 Electronics and Computer Science, Institute for Life Sciences, University of Southampton, Southampton, SO17 1BJ UK; 11 Department of Chemistry, Emory University, 1515 Dickey Drive, Atlanta, GA USA; 12 Blue Marble Space Institute for Science, 1001 4th Ave, Suite 3201, Seattle, WA 98154 USA; 13 Institute of Advanced Study, 1 Einstein Drive, Princeton, NJ 08540 USA

**Keywords:** Origin of life, Proteins, Computational chemistry, Cheminformatics, Astrobiology

## Abstract

Life uses a common set of 20 coded amino acids (CAAs) to construct proteins. This set was likely canonicalized during early evolution; before this, smaller amino acid sets were gradually expanded as new synthetic, proofreading and coding mechanisms became biologically available. Many possible subsets of the modern CAAs or other presently uncoded amino acids could have comprised the earlier sets. We explore the hypothesis that the CAAs were selectively fixed due to their unique adaptive chemical properties, which facilitate folding, catalysis, and solubility of proteins, and gave adaptive value to organisms able to encode them. Specifically, we studied *in silico* hypothetical CAA sets of 3–19 amino acids comprised of 1913 structurally diverse α-amino acids, exploring the adaptive value of their combined physicochemical properties relative to those of the modern CAA set. We find that even hypothetical sets containing modern CAA members are especially adaptive; it is difficult to find sets even among a large choice of alternatives that cover the chemical property space more amply. These results suggest that each time a CAA was discovered and embedded during evolution, it provided an adaptive value unusual among many alternatives, and each selective step may have helped bootstrap the developing set to include still more CAAs.

## Introduction

There is mounting evidence that the modern genetically coded amino acid (CAA) alphabet, used nearly universally by all living organisms on Earth, is highly optimized for a number of features including codon mapping and coverage of chemical space (*cf*. refs^1–3^, and references therein). A “chemical space” is defined as a set of compounds, hypothetical or actual, which fulfill a given set of criteria, such as molecular formula, chemical property or chemical substructure (*e*.*g*., molecules containing the α-amino acid substructure)^4–8^.

The CAAs and their corresponding codon mapping are generally believed to have been fixed by the time of origin of the Last Universal Common Ancestor (LUCA)^9^. However, the complete set of 20 likely represents the product of step-wise growth from an earlier, simpler alphabet. For example, the lower molecular weight and structurally simpler amino acids (*e*.*g*., glycine (G), alanine (A) and serine (S)) have been argued to have been made available from abiotic synthesis^10,11^, setting the stage for their incorporation into nascent biological systems^11–13^. Additionally, by most assessments, tryptophan (W) is likely to have been the last amino acid fixed into the coded set^11,14–16^, and there are computational and physical chemical data suggesting that some of the last additions were those with the greatest redox activity, which became fixed as Earth’s atmosphere became oxygenated^17^.

As there are so many possible metrics of optimality, we investigated, for the first time the optimality of the extraordinarily large number of possible coded and alternative amino acid sets (see Table 1, which is explained in more detail below) according to the metrics on which it has been claimed the amino acid set may have been selected. The 20 CAAs’ repertoire seems to reflect the requirements of providing enough structural diversity that proteins derived from these amino acids are able to define unique three-dimensional shapes^18^, and able to produce more adaptive proteins (*e*.*g*., those whose catalytic properties improve the function of the cell which hosts them, whether it be by improving turnover number, tuning it to the flux of intracellular intermediates, or by other means) when the repertoire of amino acids is enlarged^19^. These general ideas have been subsequently refined into mounting evidence for the detailed claim that a combination of size, pK_a_ and hydrophobicity seem to combine as a good first approximation of the CAA’s value to natural selection^2,3,20^.

**Table 1:** Summary of results of better XAA sets as a function of set size.

Set size (no. AAs)	# CAA Set Combinations^a^	# CAA Maximal Sets^b^	% Better XAA Sets in 10^8^ Trials
3	1,140	509	0.499
4	4,845	1,250	0.242
5	15,504	2,151	0.161
6	38,760	2,875	4.65 × 10^−2^
7	77,520	3,044	1.68 × 10^−2^
8	125,970	3,177	7.52 × 10^−3^
9	167,960	2,787	3.58 × 10^−3^
10	184,756	2,160	1.23 × 10^−3^
11	167,960	1,566	3.77 × 10^−4^
12	125,970	1,181	1.59 × 10^−4^
13	77,520	799	1.75 × 10^−4^
14	38,760	504	1.68 × 10^−4^
15	15,504	289	2.10 × 10^−4^
16	4,845	165	3.24 × 10^−4^
17	1,140	74	2.07 × 10^−4^
18	190	28	6.47 × 10^−5^
19	20	8	1.09 × 10^−5^
20^c^	1	1	6 × 10^−6^

^a^This number is derived from the formula for binomial coefficients, see SI Section 1. ^b^This is the number of maximal sets, see methods section. ^c^There is only one possible set, which is also maximal, of the 20 CAAs, and only 6 better XAA sets were found in a previous study, of which several contained CAAs^3^.

Biology produces several α-amino acids in the process of synthesizing the CAAs that are not themselves encoded (for example ornithine, diaminopimelic acid and homocysteine). Further, other amino acids are produced in the process of synthesizing various biochemicals that are also not included in the coded set (*e*.*g*., β-alanine and canavanine). It seems reasonable that several mechanisms led to the evolutionary selection of the CAAs as they were made available during the emergence of metabolism, and their incorporation into the genetic code was determined based on factors besides prebiotic availability^14,21–23^. From a different perspective, biological engineering has shown that a wide variety of amino acids can be removed, replaced or substituted in coded proteins^24,25^, though to our knowledge a complete organism-wide replacement of a CAA has not yet proven possible (*e*.*g*., compare refs^26,27^).

It has so far not been proven possible to construct an organism that can survive with less than the 20 CAAs in its total proteome^28^, and thus still not yet possible to experimentally explore metabolism(s) based on fewer than 20 amino acids, due to the high degree of interconnectivity of biological structures, components and processes^29,30^. While many modern proteins do not contain all 20 CAAs (see Figures SI1, SI2), single amino acid types have been systematically removed from specific proteins (*e*.*g*., ref.^31^), and non-biological amino acids incorporated into others (*cf*. ref.^32^). Though it appears that it would be difficult to explore the biological implications of reduced CAA sets in modern engineered organisms, chemoinformatics offers methods to explore the possible consequences of unique CAA set composition trajectories during pre-LUCA biochemical evolution. We herein used computational approaches to estimate the adaptive value of potential coded α-amino acid subsets which biology might have explored during biochemical evolution.

The CAAs are distinguished from theoretical alternatives by their properties as a set, specifically their coverage of chemical space^3,33^. We therefore assumed that, in the growth of the amino acid alphabet, individual α-amino acids were selected based not only on their own intrinsic physicochemical properties but also on the way that complemented a pre-existing set. We then investigated an exhaustive set of possible subsets, evaluating them in terms of their coverage of chemical space relative to theoretical α-amino acid sets sampled from a larger virtual library of 1913 amino acids^3^ constructed using molecular structure generation software^34–36^. Although other factors, including biosynthetic availability, could have contributed to shaping the growth of the modern near-universal CAA alphabet, we sought to establish whether the CAAs can be distinguished as optimal using a minimal set of parameters, namely size, hydrophobicity and pK_a_^3^.

## Materials and Methods

We used a reference library consisting of the 20 CAAs and 1893 other theoretically possible, computationally constructed α-amino acids from a previous study^3^. We refer to this set of molecules as the *xeno amino acids* (XAAs). Given that the library from which the XAA sets were selected includes the 20 CAAs, some XAA sets also contain CAAs. To describe the properties of the molecules in the computed sets, we used the same descriptors previously reported: van der Waals volume (V_vdw_), logarithmic acid dissociation constant (pK_a_, considered specifically over the range from 2–14 here) and partition coefficient (logP), which were selected based on their ability to characterize the functional chemistry space of α-amino acids (*cf*. refs^3,37^). V_vdw_ is simply a measure of the volume of space occupied by the amino acid, which is expected to play a role in mediating steric interactions. LogP describes the affinity of a molecule to a hydrophilic or hydrophobic solvent. In the context of protein structure, this is an important factor in protein folding, and is essential for the heterogeneous nature of protein surface potentials, which is essential for catalysis. pK_a_ describes the pH at the mid-point of a proton transfer by the amino acid side chain, which influences the charged state of an amino acid residue. This in turn affects a host of interactions within and among proteins and their substrates.

While thousands of additional descriptors exist^38^, we selected what we considered to be fundamental properties that define molecular interactions. This selection was made to minimize bias introduced by considering instead the properties through which we functionally characterize amino acids in modern biochemical contexts. Additionally, these properties are reliably predicted and quantified through chemical property prediction software, an important consideration given the theoretical and computational nature of our data set. This analysis is exploratory, requiring broad, and simplified, choices of molecular descriptors. A previous publication^20^ offers a specific investigation that justifies the descriptors relating to size, pK_a_ and hydrophobicity, particularly in the light of older work^39^ that has been built on productively^40,41^. It is striking that such simple metrics are able to classify amino acid chemical space so effectively^2,3^.

We used the definition of optimality that was previously introduced to test the CAA alphabet, as described in Ilardo *et al*.^3^, namely that more “optimal” sets are those that have *broader range and/or evenness* of coverage of chemical space. “Better” or “more optimal” sets are those that cover chemical space in the three descriptor categories (V_vdw_, pK_a_ and logP) both more broadly and evenly than a comparison set. For calculation of range, evenness and coverage, please see Section 1 of the supporting information (SI) and for further discussion see refs^2,34^.

We first generated a comprehensive list of all possible *k*-subsets of the CAAs ranging in set size from three (the smallest size that allows calculation of evenness) to 19 amino acids (as 20 was a previously calculated benchmark^3^) and their range and evenness values in the dimensions of size, pK_a_ and hydrophobicity. A part of this list showing the computed values for set size 19 is given in Table SI1. From these, we computed sets of maximal coverage (hereafter referred to as *maximal sets*), defined as sets for which there exists no other set of the same size that is “better,” with respect to range and evenness in coverage of chemical property space with respect to V_vdw_, pK_a_ and logP. These maximal sets are maxima of the partial order introduced by the six dimensions (range and evenness in size, pK_a_, and hydrophobicity) of the *k*-subsets (*k* > 2) of CAAs^42^. Partial orders are a frequently used concept in chemistry whenever there is no total order available^43,44^. Section 2 of the SI gives a formal definition of our partial order and a brief introduction to Hasse diagrams, which can be used as graphical representations of partially ordered sets. Figure SI3 shows a Hasse diagram of the 19-subsets of the CAAs.

To quantify the optimality of the maximal subsets of CAAs, we needed an appropriate comparison. As the number of combinatorially possible sets of the XAAs that can be constructed grows exponentially with set size (see Figure SI4), we used a statistical sampling approach and generated 10^8^ random sets for each set size selected from the XAA reference library. By repeatedly sampling different set sizes (10^7^–10^9^), we were able to establish that a sample size of 10^8^ sets provides asymptotic results (*i*.*e*., larger sample sizes do not produce different results) that are computationally tractable (*e*.*g*., returning results on the timescale of ~ 1 day using our available computational resources; sampling 10^9^ sets took on the order of a few weeks). Each of these XAA sets was then compared to the maximal CAA subsets, which are again those for which the range and/or evenness in one of the three optimality criteria are not surpassed by another CAA set for that set size. In each comparison the set with more optimal chemical space coverage (as previously described) was designated a “better set”. We recorded the number and composition of each better set identified from the XAA sets. In order to gain high certainty on the computational correctness of our results, the algorithms were implemented independently once in Matlab and once in Python. They were run on a Hewlett-Packard Pavilion Windows PC with an Intel(R) core(™) i7-7700HQ 2.8 GHz CPU 32 GB RAM, using up to eight cores in parallel.

Z-values offer a way to compare results to a normal population. A z-value is **a** measure of how many standard deviations below or above the population mean a raw score is. Negative Z-values fall to the left of the normal distribution curve and positive ones to the right. The Z-value formula for a sample is:

$${\rm{z}}=({\rm{x}}-{\rm{\mu }})/{\rm{\sigma }}$$

where x is the measured value, μ is the mean and σ the standard deviation. Z-values shown were computed using Microsoft Excel.

For the extant protein analysis, we considered the 554,514 proteins annotated in the manually curated Swissprot database^45^ as of 2017, which included a total of 198,500,435 unique amino acid residues (Figure SI1). Of the proteins containing less than 20 unique CAAs (184,929) we identified those CAAs that were absent. Proteins with multiple absences contribute to each of the relevant counts. Figure SI2 shows the proportion of database protein sequences lacking each CAA.

## Results

In order to test the adaptive value of the CAAs at smaller set sizes, we first measured the number of better sets, as described in the methods section, for each set size. The results, listed in Table 1, highlight the significant adaptive properties of the CAAs at even the smallest set size.

**Figure 1:**
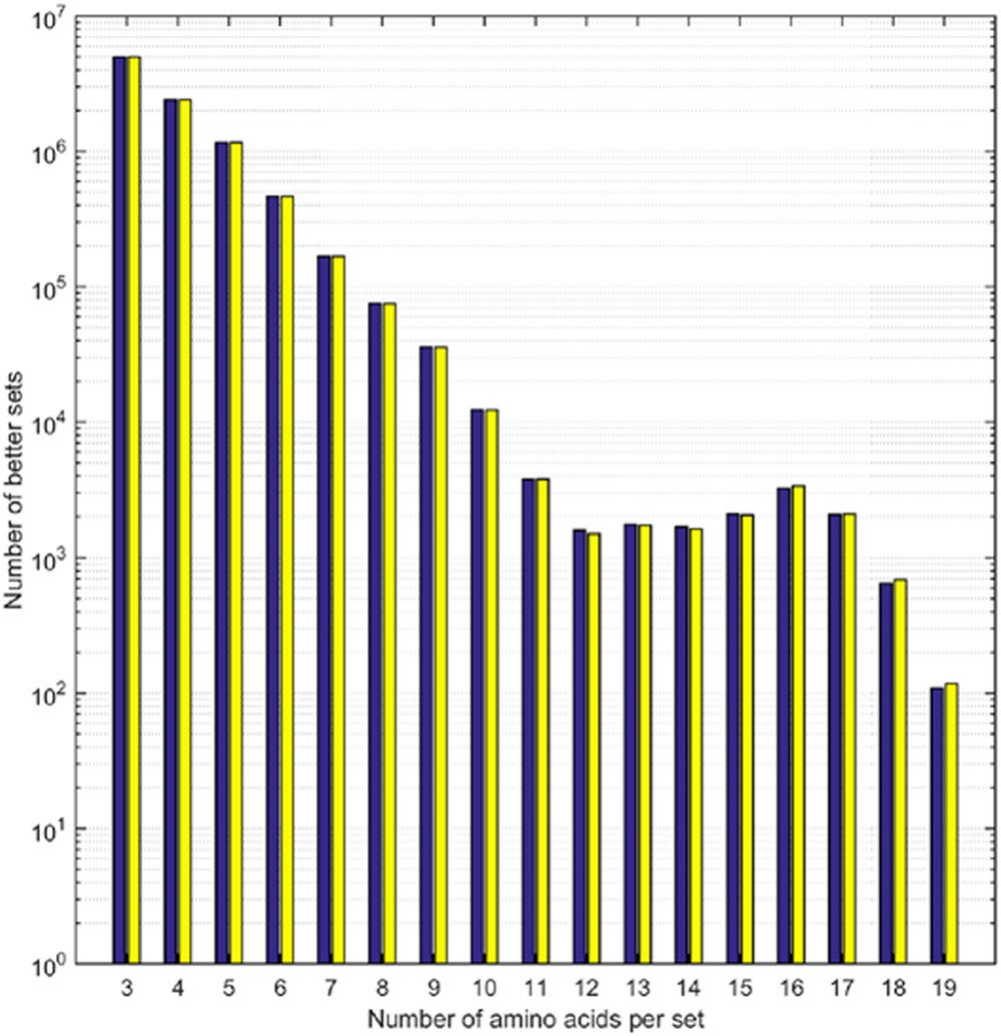
Semi-log plot showing the results of two 10^8^ samplings (yellow and blue bars) for better XAA sets of a given set size (shown on the x-axis) from the XAA library. The number of better XAA sets decreases approximately logarithmically with the exception of sets of size 13 to 18.

For example, we find that already for a set size of three amino acids only about 5% of random XAA sets are “better” than at least one of the maximal CAA sets. Interestingly, the number of better sets roughly follows a trend of logarithmic decrease with set size (Fig. 1), consistent with the results reported in Ilardo *et al*.^3^.

There is a slight anomaly between set sizes of 13–18. This deviation from monotonic exponential decline in better sets was intriguing, and we thus conducted further tests to distinguish between the possibility that it was an artifact of the 10^8^ sample size (*i*.*e*., a sampling error since there are generally vastly more combinations possible than tested) versus other possible explanations (*e*.*g*., the chemical similarity of some CAA’s such as Ile and Leu, which make the “landscape of the better” relatively flat in places).

Specifically, to do this we repeated the calculation with the extremely rare better 20-amino acid member XAA sets 1 and 2 from Fig. 3 of Ilardo *et al*.^3^. The shape of the deviation was consistent (Figure SI5), though interestingly the better XAA sets were found to have fewer better-set combinations than strict CAA sets in random samples, suggesting there may be theoretical CAA better sets which evolution did not find. Regardless, this result suggests that the deviation is indeed a function of the properties of the CAAs (and those of the members of the better XAA sets) *as ensembles* relative to the properties of the entire XAA library’s properties.

**Figure 2:**
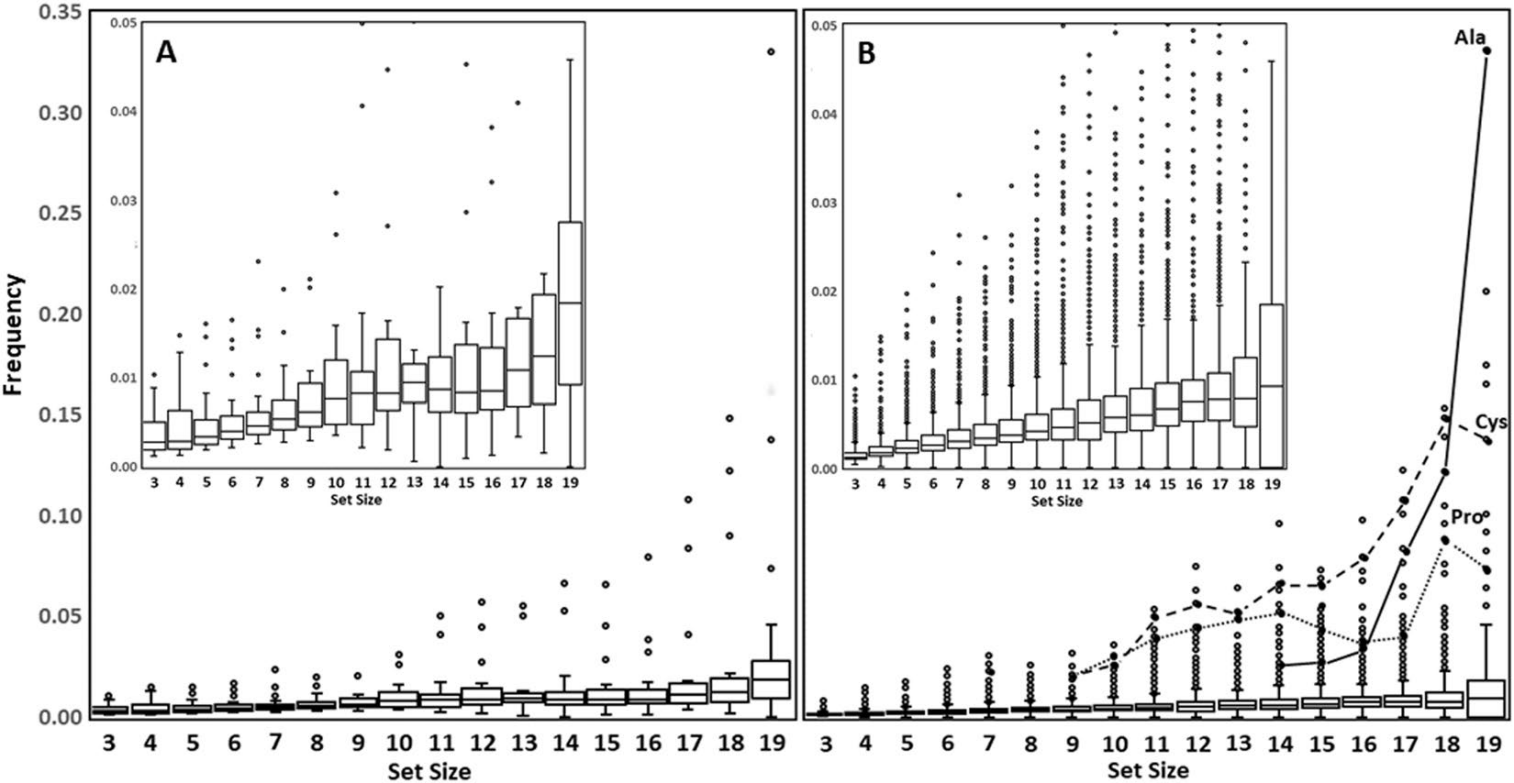
Box plots showing the relative frequency of (**A**) CAAs and (**B**) XAAs in better sets. Boxes extend from the lower to upper quartile values of the data, with a line at the median, and whiskers extending to contain 95% of the data. Zoomed insets help to show that the comparison of (**A**,**B**) reveals that the median values for maximal CAA sets are always higher than those of the corresponding XAA set sizes. In (**A**) all top outlier data points represent Met in set sizes 16–19. The connected data points in Figure B highlight the anomalously high frequency of the CAAs, Ala, Cys and Pro, in better XAA sets of larger set size.

**Figure 3:**
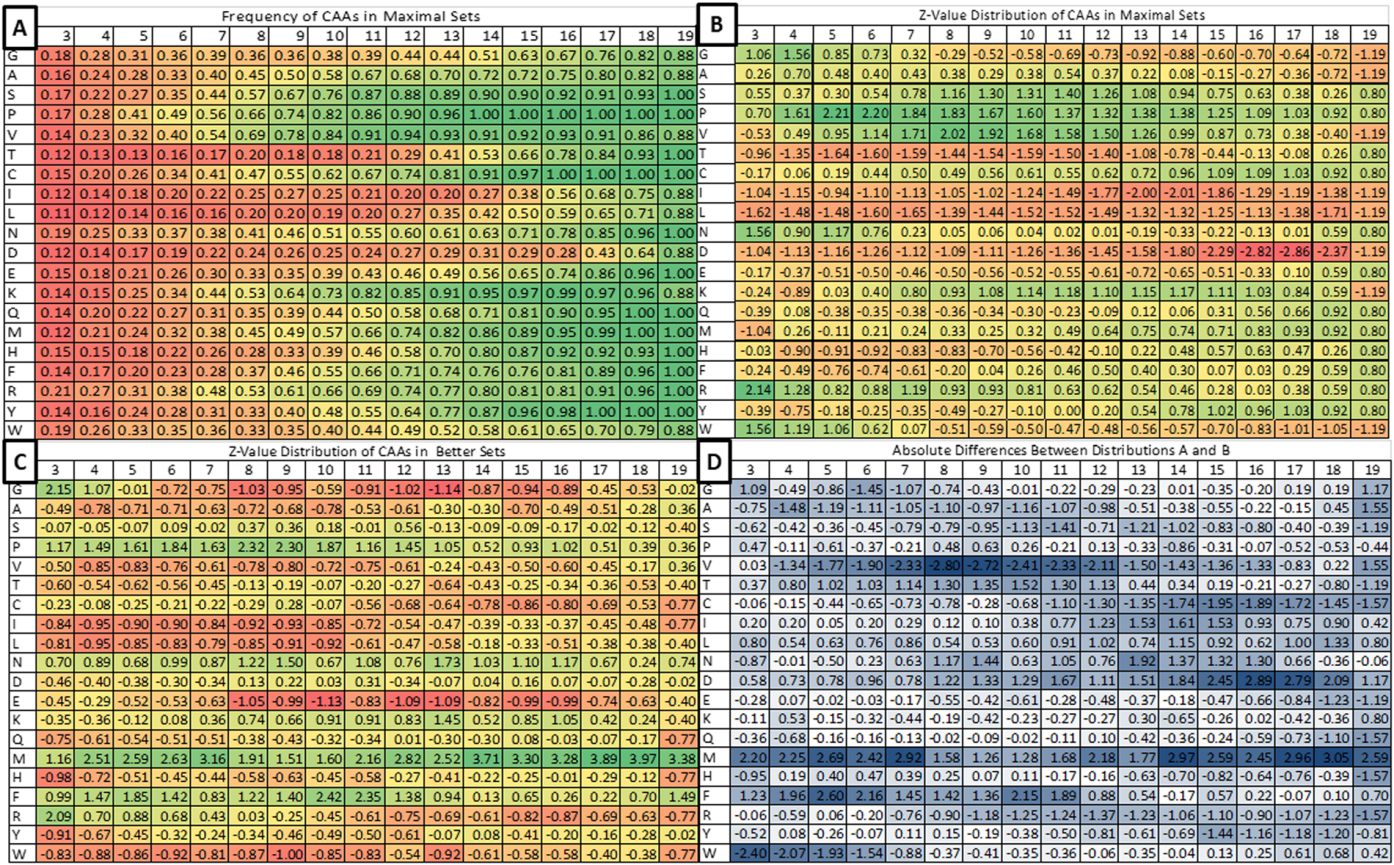
Relative frequency at which the individual CAAs are found in maximal CAA and better XAA sets. (**A**) shows the raw relative frequency of occurrence of the CAAs in maximal sets. (**B**) shows the Z-value for the frequency distribution shown in (**A**). In (**C**), green corresponds to a particular CAA occurring at high frequency relative to the other CAAs in sets, while red corresponds with low frequency. In (**D**), the absolute difference between the relative frequencies (Z-values) shown in (**B**) and (**C**) highlight areas where the relative frequencies of a particular amino acid vary between maximal and better sets, possibly highlighting CAAs having different importance depending on the context in which they are compared. Dark blue indicates a large difference between the frequency of a particular CAA in maximal CAA vs. better XAA (*e*.*g*., those selected from the total XAA pool) sets. The direction of the bias can be determined by referring to panels B and C. Rounded raw values are shown for reference in each data cell.

We further examined whether the adaptive contribution of the CAAs to XAA better sets could be detected at the level of individual amino acids. We measured the frequency of representation of CAAs in better sets also containing XAAs and compared this to the frequency of non-CAA XAAs in those better sets. The distribution of these values are shown in the box plots in Fig. 2.

Some of the CAAs are noticeably statistically overrepresented even at the smallest set sizes, and this adaptive advantage increases dramatically as set size grows. In Fig. 2, Met, Pro and Phe consistently rank among the most frequent CAAs in better sets, with Gly being abundant in set sizes of 3 and 4. By set sizes of 17, at least one CAA is represented in almost 30% of better sets, and many contain more than one (Figure SI6).

Lastly, we looked at the individual CAAs in the maximal and better sets to determine, whether particular amino acids exert a larger influence than others on our interpretation of adaptive properties for the subsets. Figure 3 shows the distribution of the CAAs in the better and maximal sets.

## Discussion

It is unknown which set of amino acids was used before the advent of LUCA, in which the modern CAA set had already been adopted. However, by computing all combinations, we can explore the adaptive potential of many possible subsets. Assuming that early in CAA selection, each CAA addition created, in effect, a “negative chemical property space,” in analogy with the term “negative space” as used in the art world – each occupation of the real implies how the occupation of the potential could be occupied. This negative space is then more likely to be filled by a new amino acid that would make the resulting new CAA set more adaptive, then somewhere in the CAA maximal sets (as defined in the Methods section) computed here there are likely to exist representations of true earlier (however weakly encoded) alphabets. The number of XAA sets identified that appear more adaptive than even one such maximal CAA set is therefore strikingly low. This suggests the CAAs contain members that are directionally optimal with respect to their chemical properties, regardless of the biosynthetic pathways that produce them.

We can presently only infer the composition of amino acid alphabets that life used pre-LUCA. Therefore, we also examined the frequency of individual CAAs in better XAA sets, as this can give an indication of which amino acids contribute to the adaptive properties exhibited by smaller (<20) amino acid alphabets of different sizes. We examined this frequency in two contexts, the frequency of particular amino acids in maximal sets, which only contain CAAs (Figs 3A and 3B), or better sets, where the possible set members (and therefore the context in which they appear adaptive) include all XAAs (Fig. 3C). Interestingly, there are differences in how adaptive the CAAs appear relative to the context in which they are examined (Fig. 3D). For example, Trp occurs infrequently in small size sets (n ≤ 10) selected from all XAAs, but occurs frequently in small-sized maximal sets selected from the CAAs. One interpretation is that although W expands the chemical space of CAAs, many other, theoretically possible amino acids could have provided a similar advantage. Conversely, Met appears to be advantageous in small set sizes compared to the possibilities within the XAAs. However, it is not until larger set sizes that Met begins to become adaptive when looking at the coded set. This could indicate that Met does not offer an enormous adaptive advantage to the encoded set as a whole, however, there are very few possibilities within the XAA library that have similar chemical properties to Met, and therefore Met is uniquely advantageous to encoded sets.

For the contribution of the CAAs to making sets maximal (Fig. 3A), several interesting trends are evident. For example, Gly, Asn, Arg and Trp play large roles in making sets maximal at small set sizes, while several CAAs (*e*.*g*., Ser, Pro, Val and Lys) play large roles in intermediary set sizes, and others in relatively larger ones (Cys, Gln, Met, His, Phe and Tyr). The last cohort roughly corresponds to various explanations offered for the order of incorporation of the CAAs (*e*.*g*.,^11,13,17^) to the modern CAA set. For the contribution of CAAs to constructing better XAA sets (Fig. 3C), some of the same trends are evident, *e*.*g*., that of Gly for small sets and that of Met for larger ones, as well as the general utility of Pro, Asn and Phe.

The difference of these contributions is shown in Fig. 3D, which highlights the importance of the occurrence of other CAAs to make any given CAA appear to be a large contributor to set adaptiveness by these criteria. Here, white and dark blue regions highlight instances where another amino acid is likely required to bring out the adaptive value of an added CAA. These likely occur when a given CAA has some property that is an outlier in property space, which underscores our general model.

The structures of the XAAs represented most frequently in better sets, accumulated over all set sizes, are shown in Figure SI7 together with their frequencies. Some appear frequently independent of set size, in particular the two dehydroproline analogues (Figure SI7a,c). Others, like 2-amino-3,5-hexadienoic acid (Figure SI7e), are only frequent at larger set size (in this case 14–19), while still others like 3-aminoleucine are only frequent among small (3–9) set sizes. The nine structures shown in Figure SI7 are presented using an arbitrary frequency cut-off simply for the sake of exposition, but the frequency with which certain structures appear may point to the potential use of these similarity metrics to introduce novel XAAs into coded proteins.

As can be seen in Figure SI5, the probability of finding random better sets depends on sampling size and becomes asymptotic in the region around 10^7^ sampled sets. At low-end sampling (10^6^ trials and below), frequencies greater or lesser than the true average can be found. Frequencies for 10^7^ and 10^8^ sample sizes are almost indistinguishable. The general logarithmic decline in frequency of better sets, as well as the transient deviations in this trend at larger set sizes, is evident for both the CAAs (see Fig. 1), as well as for XAA sets 1 and 2 from Ilardo *et al*.^3^ We believe this deviation comes simply from the nature of the possible set compositions as sets grow in size, *i*.*e*. the intrinsic structural possibilities and the properties they define, as well as the criteria by which optimality is defined here. These deviations may represent instances where there is the greatest opportunity for the incorporation of novel functionality, though by this point such opportunities are severely circumscribed by previous selections.

That Ile and Leu should appear to have a cooperative effect on fitness is at first glance surprising given their structural similarity. Indeed, these two CAAs score as the most similar pairwise CAAs according to many metrics^46–49^, and among those most subsitutable for each other in modern proteins according to the work of Yampolsky and Stoltzfus^50^.

Nevertheless, their V_vdw_, pK_a_, and logP values all vary slightly, therefore although we would expect them to have very similar adaptive value according to our metric, we also expect them to be different. This is in fact what is reflected in the heat maps shown in Fig. 3. We further tested why these two might be dissimilar enough to be co-adaptive numerically by simply subtly altering the CAA set. We found that despite their similarity, both Ile and Leu improve the evenness of the natural set, *e*.*g*., removing either lowers the evenness score. We also checked doubling Ile and Leu individually (*e*.*g*., having two instances of each, to allow them to weight their local numerical space) as psuedo controls, and this gave worse evenness scores than the both together or the omission of either. Although neither Ile nor Leu affects the range in any property dimension, the space is such that even the small amount of calculated difference in their descriptor values improves over all evenness, mainly on the basis of the LogP dimension coverage. This subtle difference is supported by solubility data, for example the Merck index^51^ lists the solubility of L-Leu in water as 22.7 g/L at 0 C while while that of L-Ile is 37.9 g/L. Thus, there are subtle but significance in the chemical properties of these two amino acids that may allow them to be collectively adaptive despite their apparent structural similarity.

Ile and Leu are thus not identical (to think so is perhaps an example of the sort of human bias one needs to be wary of), and the presence of two similar CAAs in a coded set may be the sort of legacy one would expect from a fuzzy primordial code. Further, it is possible that in an earlier, more promiscuous preiod of biochemical evolution several there were “isomeric twins” or otherwise similar set members that may have buffered against damaging mutations to physico-chemically dissimilar amino acids. For example, changing the first position of four of the seven Leu codons converts it to Ile. Once protein complexity increased past a certain threshhold, selection operated on finer structural features.

There are of course likely other determining factors that were involved in the selection of life’s biochemical toolkit beyond those that can be addressed in this type of numerical evaluations. For example, shape and steric properties are important for protein folding. Nevertheless, the number of amino acids with similar size and hydrophobicity values is fairly small, depending of course on the cutoff used for evaluating similarity.

It is noteworthy that for XAA set 1 no better sets are found beyond a set size of 16 for 10^6^ trials nor beyond a set size of 18 for 10^7^ trials, and for XXA set 2 none beyond a set size of 17 for samplings from 10^6^ to 10^8^ trials, which actually outperforms the actual CAA set at certain set sizes. We believe the idea that there might be many more optimal sets than the CAAs should perhaps be tempered first by the fact that one of these better XAA sets contains two CAAs, and second that neither of them has been analyzed with respect to their potential biosynthetic relationship or various other factors. For example, Gly, Ser, Ala and Cys belong to a fairly tight biosynthetic clique, as do Phe, Tyr and Trp, Asp and Asn, Glu and Gln, and to an extent the branched chain AAs.

This analysis does not take into account biosynthetic pathways and that “network closure,” the notion that all processes inside a cell should be linked and share common resources for efficient coordination of metabolism, may have been important in the adaptive evolutionary construction of biochemistry^52^. It would be an interesting exercise to see which types of hypothetical metabolic networks can be constructed among the XAA better sets. However, as XAA better sets are already rather rare among the entire cohort, it seems likely that the addition of such a constraint would only make the emergence of the actual CAA set appear more adaptive and predisposed.

As the full diversity of possible enzymatic transformations using CAA comprised proteins is unknown, and that of potential XAA comprised peptide polymers completely unknown, it is impossible to make very strong statements about the completeness of coverage of catalytic mechanism space by modern biochemistry. However these sorts of relationships also could have affected the search space biology explored during the development of coded sets.

It should also be noted there is some debate about the mechanism of genetic code evolution and the role of selection in its origin (see for example refs^53–59^), nevertheless since it seems unlikely all of the CAAs were available from prebiotic synthesis^14^, and thus some may have been adopted into the code after organisms developed the ability to biosynthesize them. Their addition, or non-addition, would then be selectable. There are of course alternative hypotheses one could entertain, for instance that organisms were able to biosynthesize all of the CAAs before any part of the code was canonized, but the stepwise expansion and rewiring of a more primitive code remains a compelling possibility.

One last note should be added here. The XAA library used here is the simplest computed by Meringer *et al*.^34^, however, the larger libraries computed therein cover chemical property space based on ways we think justify the use of this smaller library in the present study. There are always more structural variations of larger amino acids than of smaller ones, and the coverage of chemical space becomes very dense for larger molecules because the nuances of structural diversity become subtle. The population of the search space with respect to the properties of the targets could alter the landscape of these results, but we do not feel it would do so qualitatively. The smaller amino acids, for which there are fewer isomers, would still occupy a corner of property space; and the larger ones would occupy regions that are highly redundantly occupied. Using the exhaustive list of alternatives would thus also tend to weight the smaller amino acids, and de-weight amino acids of higher molecular weight. This would tend to underscore the point that the CAAs are positioned in property space in such a way that they are likely selectional outcomes regardless of search blank.

## Conclusions

Our analysis suggests that stepwise expansion of the CAA repertoire proceeding through the present CAAs represents a trajectory that became increasingly adaptive relative to hypothetical sets that can be constructed from XAAs. These results pose a number of other questions: why then did biology (with the two known infrequently occurring exceptions of selenocysteine and pyrrololysine) stop exploring chemical space? Is it because property space was already well-explored based on these principles? Our results suggests that this is indeed the case - the possible chemical space both limits and directs the explorable space. This analysis suggests that once evolving organisms acquired one or more amino acids from the modern CAA set, the organisms encoding them would also be poised on a trajectory to incorporate still other modern CAAs, *i*.*e*., the fitness landscape would have steered organisms to fill amino acid selection in roughly the same way. We might then expect organisms on other Earth-like planets to use similar amino acids.

## Supplementary information

Supporting Information

## Data Availability

The datasets generated during and/or analyzed during the current study are available from the corresponding author on reasonable request. The compound library of 1913 amino acid structures as SD file can be downloaded from www.molgen.de/data/AACLBR.sdf.zip.
