# Supplementary material for: Adaptive Properties of the Genetically Encoded Amino Acid Alphabet Are Inherited from Its Subsets
Source: Sci Rep. 2019 Aug 28;9:12468. doi: 10.1038/s41598-019-47574-x (PMC6713743; doi:10.1038/s41598-019-47574-x)
Supplement: Supplementary file 1 — Supporting Information [file 41598_2019_47574_MOESM1_ESM.docx]

Supporting Information for **Adaptive Properties of the Genetically Encoded Amino Acid Alphabet Are Inherited from Its Subsets**

Melissa Ilardo^1^, Rudrarup Bose^2^, Markus Meringer^3^, Bakhtiyor Rasulev^4^, Natalie Grefenstette^5^, James Stephenson^6, 7^, Stephen Freeland^8^, Richard J. Gillams^9, 10^, Christopher J. Butch^9, 11, 12^ and H. James Cleaves II^9, 12, 13*^

**1 Range, Evenness and Coverage**

Let *p*_1_ ≤ *p*_2_ ≤ ... ≤ *p_n_* be the sorted values of a property *P* ∊ {pK_a_, V_vdw_, logP} for a set *A* of *n* amino acids. Then the range of *A* with respect to *P* is defined as the difference of maximum and minimum values:

ρ(*A*, *P*) = *p_n_* - *p*_1_

and evenness of *A* with respect to *P* is computed as the variance of differences of successive property values:

ε(*A*, *P*) = Var {*p_i_* - *p_i_*_-1_ | 1 < i ≤ n}.

Better coverage means higher range and lower evenness. Using the above equations we define the coverage of a set *A* of amino acids as the 6-tuple:

(*A*) = (ρ(*A*, pK_a_), ρ(*A*, V_vdw_), ρ(*A*, logP),- ε(*A*, pK_a_),- ε(*A*, V_vdw_),- ε(*A*, logP))

composed of range and negative evenness values in the three considered properties, charge, size and hydrophobicity.

**2 Partial Order and Hasse Diagrams**

Using the above formalism we can say that a set *B* is better than a set *A* if 𝜅(*B*) > 𝜅(*A*), where the 'greater' relationship for two 6-tuples *a*=(*a*_1_,...,*a*_6_) and *b*=(*b*_1_,...,*b*_6_) is defined in the following natural way:

*b* ≥ *a* : ⇔ *b*_1_ ≥ *a*_1_ ˄ … ˄ *b*_6_ ≥ *a*_6_,

*b* > *a* : ⇔ *b* ≥ *a* ˄ *b*_6_ ≠ *a*_6_.

This relationship defines a partial order on the set of 6-tuples, and via the mapping this ordering can be used for sets of amino acids. In contrast to totally ordered sets, in a partially ordered set (short *poset*) not every pair of set elements needs to be in a relationship. This fits perfectly our use case. For instance the first two sets of Table SI1, where once proline (P) and once alanine (A) is removed from the CAA set *C*, we neither say that *C*\{P} is better than *C*\{A}, because:

ε(*C*\{P}, pK_a_) = 0.034434 > 0.024547 = ε(*C*\{P}, pK_a_),

nor the opposite, because:

ε(*C*\{P}, V_vdw_) = 31.71294 > 33.26546 = ε(*C*\{P}, V_vdw_).

A poset can be visualized using a directed acyclic graph (DAG) where the vertices represent the set elements and edges represent relationships between set elements, i.e. for each relationship *a* < *b* there is an edge from vertex *a* to vertex *b*. The transitive reduction of this DAG is another directed acyclic graph with the same vertices and as few edges as possible, such that for each relationship *a* < *b* there is a directed path from vertex *a* to vertex *b* in the reduction. A Hasse diagrams is a drawing of this transitive reduction such that all edges have upward direction. Figure SI3 shows a Hasse diagram obtained from the data given in Table SI1.

**3 Basic Combinatorics**

The binomial coefficient is defined as ${{}_{n}C}_{k}=\binom{n}{k}=\frac{n!}{k!\left( n-k \right)!}$. It equals the number of *k*-subsets out of a set of *n* distinct items. Often it is called combinations of *k* out of *n* items, or just “*n* choose *k*”.

**4 Probabilistic Combinatorics**

Basic combinatorics can be used to calculate certain probabilities. We used them here to calculate the probability that a randomly chosen *k*-subset out of *n*=1913 XAAs contains exactly *m* CAAs. It is $P\left( k,m,n \right)=\binom{20}{m}\binom{n-20}{k-m}/\binom{n}{k}$.


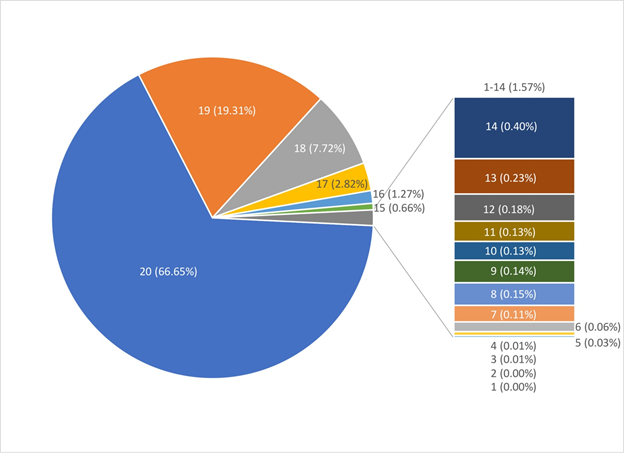


**Figure SI1.** The percentage of proteins (in brackets) from a global search of Swissprot containing different numbers of the CAAs, demonstrating that while the majority of extant proteins use the full CAA complement, a significant fraction do not.


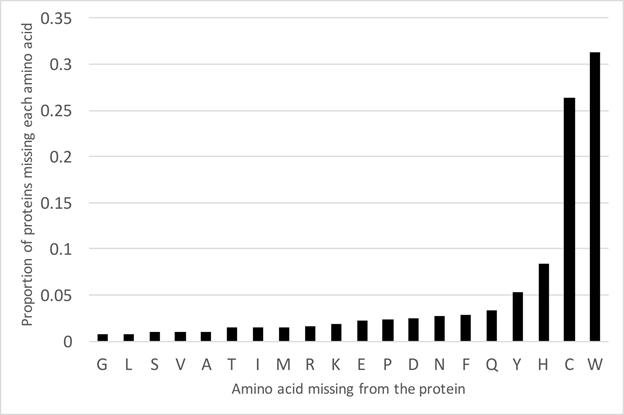


**Figure SI2.** Proportion of proteins missing each amino acid for proteins with at least one of the CAAs absent. Bars show the abundance of each amino acid as a proportion of all amino acids in all protein sequences deposited in Swissprot (198,500,435 total amino acid residues). Variations below or above 0.05 show a relative enrichment or depletion, respectively, of that amino acid.

**Table SI1**. Range and evenness values of size, pK_a_ and hydrophobicity for the subsets containing 19 CAAs.

| **CAA**  **not in Subset** | **V_vdw_**  **Range** | **V_vdw_**  **Evenness** | **pK_a_**  **Range** | **pK_a_**  **Evenness** | **logP**  **Range** | **logP**  **Evenness** |
| --- | --- | --- | --- | --- | --- | --- |
| P | 122.335 | 31.71294 | 2.765 | 0.034434 | 3.20 | 0.023028 |
| Y | 122.335 | 33.26546 | 2.765 | 0.024547 | 3.20 | 0.014428 |
| W | 101.805 | 21.33779 | 2.765 | 0.016722 | 3.11 | 0.015042 |
| F | 122.335 | 38.74661 | 2.765 | 0.016922 | 3.20 | 0.016840 |
| N | 122.335 | 32.25050 | 2.75 | 0.016960 | 2.91 | 0.014536 |
| Q | 122.335 | 32.08441 | 2.765 | 0.017342 | 3.20 | 0.017284 |
| C | 122.335 | 37.30384 | 2.765 | 0.023272 | 3.20 | 0.014962 |
| M | 122.335 | 31.87304 | 2.765 | 0.017339 | 3.20 | 0.023873 |
| S | 122.335 | 40.01143 | 2.765 | 0.016809 | 3.20 | 0.017040 |
| T | 122.335 | 30.81285 | 2.765 | 0.018453 | 3.20 | 0.013940 |
| D | 122.335 | 31.05583 | 2.765 | 0.016897 | 3.20 | 0.014140 |
| E | 122.335 | 34.04894 | 2.765 | 0.017122 | 3.20 | 0.014306 |
| K | 122.335 | 43.05824 | 2.765 | 0.024050 | 3.20 | 0.013906 |
| H | 122.335 | 30.57229 | 2.765 | 0.023317 | 3.20 | 0.017340 |
| R | 122.335 | 31.46327 | 2.495 | 0.017050 | 3.20 | 0.015517 |
| A | 122.335 | 47.78322 | 2.765 | 0.016739 | 3.20 | 0.015517 |
| V | 122.335 | 35.80894 | 2.765 | 0.016733 | 3.20 | 0.023340 |
| L | 122.335 | 30.72936 | 2.765 | 0.016795 | 3.20 | 0.016940 |
| I | 122.335 | 30.55793 | 2.765 | 0.018200 | 3.20 | 0.013917 |
| G | 104.763 | 25.71333 | 2.765 | 0.017642 | 3.20 | 0.014873 |


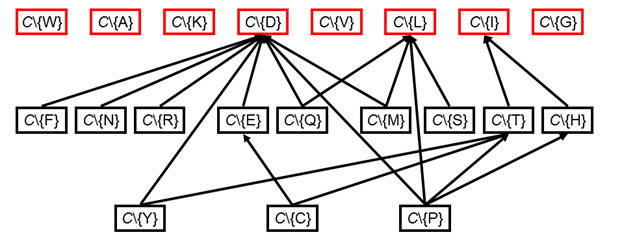
**Figure SI3.** Hasse diagram of the 19 CAA-containing subsets. The CAA missing from the set’s single letter code appears in brackets. An arrow from *A* to *B* means that *B* has better coverage than *A*. In the top row the eight maximal subsets are shown. Five of them are isolated maxima.


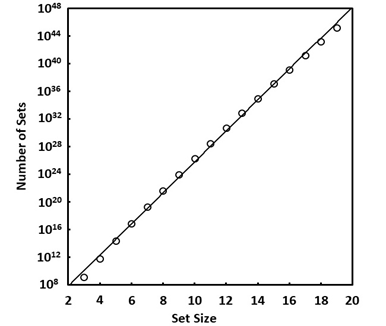


**Figure SI4.** Number of possible combinations of XAA sets vs. set size. The data can be fit with the equation y = 1998.4e^5.1702x^ with an R² value of 0.9983.


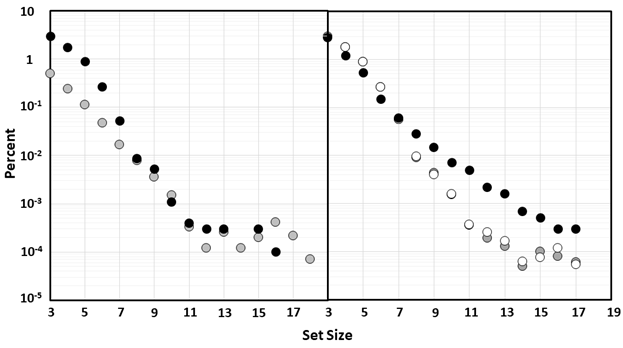


**Figure SI5.** Results of random searches for better sets than better XAA sets 1 (left, 10^6^ and 10^7^ trials) and 2 (right, 10^6^, 10^7^ and 10^8^ trials) from Figure 3 of Ilardo et al. (2015). Color coding by trial number: 10^6^ (black); 10^7^ (grey); 10^8^ (white) trials. For XAA set 1 no better sets were found for 10^6^ trials for set sizes of 17-19 or 10^7^ trials for set size 19, and in the right hand panel, small set size values (*e.g.*, 3-11) are often closely superposed, and no better sets were found for any trial size for set sizes of 18 or 19.


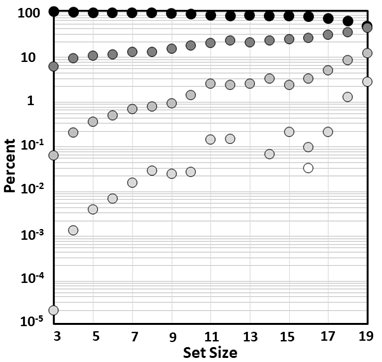


**Figure SI6.** Percentage of better XAA sets containing CAAs as a function of set size. Black markers: sets containing no CAAs, increasingly pale grey markers: sets containing 1-4 CAAs. Note that sets containing four CAAs are rare, and no sets containing three were found for set size 13. For reference, the probabilities of including *m* CAAs randomly in a set of *k* are given in Table SI2.

**
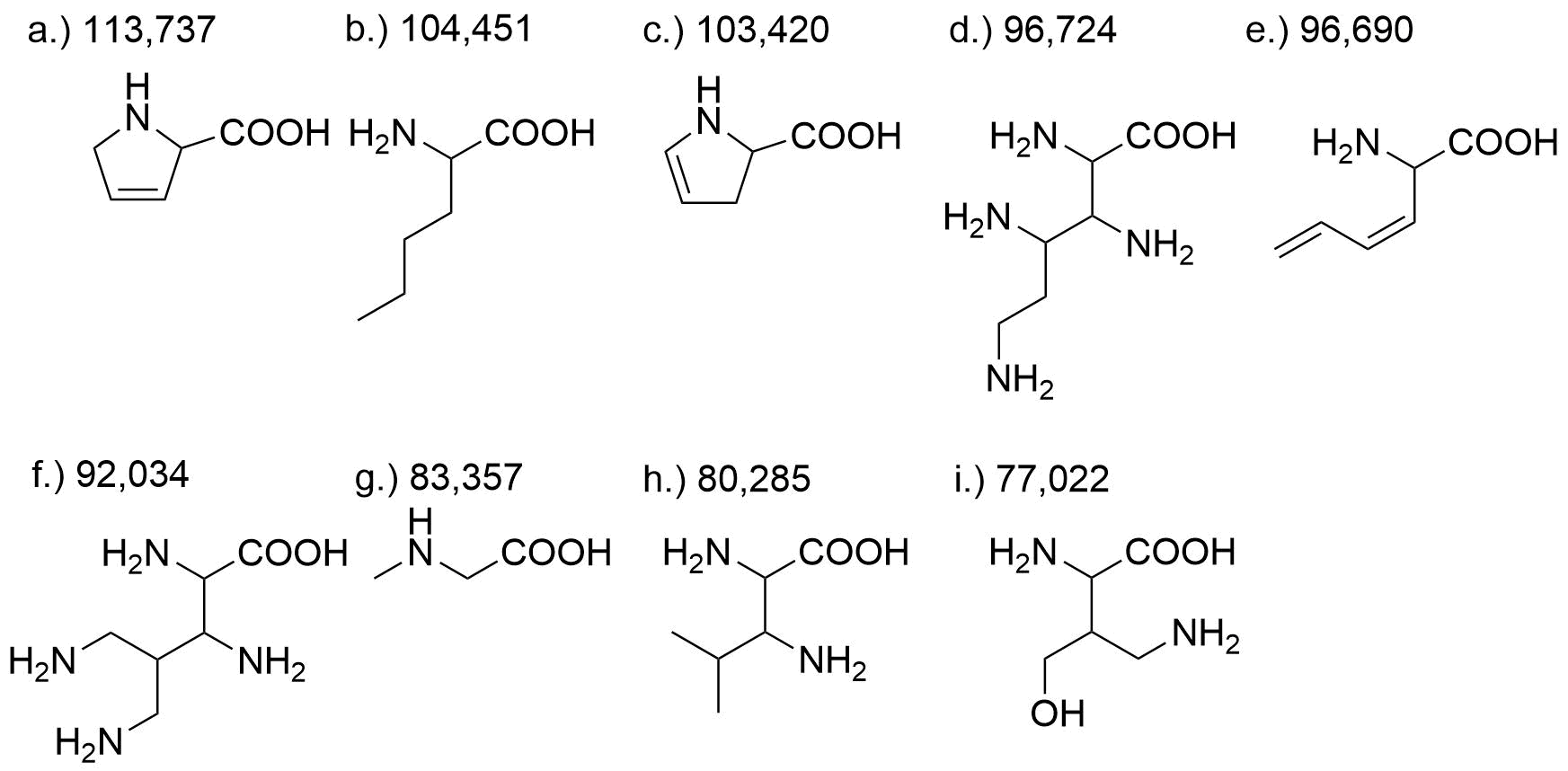
**

**Figure SI7.** The structures of the XAAs most frequently represented in better sets, accumulated over all set sizes, together with their frequencies.

**Table SI2**. Probabilities of including *m* CAAs randomly in a set of size *k*. These values are calculated from the formula given in Section 2 of this SI with *n* = 1913.

| *k* | *m* = 0 | *m* = 1 | *m* = 2 | *m* = 3 | *m* = 4 |
| --- | --- | --- | --- | --- | --- |
| 3 | 0.9689463 | 0.03074393 | 0.0003087393 | 9.785715e-7 | 0.000000 |
| 4 | 0.9588003 | 0.04058414 | 0.0006116594 | 3.879447e-6 | 8.709799e-9 |
| 5 | 0.9487553 | 0.05022527 | 0.0010098201 | 9.612249e-6 | 4.318399e-8 |
| 6 | 0.9388102 | 0.05967014 | 0.0015004403 | 1.905321e-5 | 1.284656e-7 |
| 7 | 0.9289643 | 0.06892157 | 0.0020807889 | 3.304588e-5 | 2.972381e-7 |
| 8 | 0.9192165 | 0.07798231 | 0.0027481843 | 5.240182e-5 | 5.894858e-7 |
| 9 | 0.9095659 | 0.08685510 | 0.0034999936 | 7.790129e-5 | 1.052163e-6 |
| 10 | 0.9000117 | 0.09554264 | 0.0043336317 | 1.102939e-4 | 1.738868e-6 |
| 11 | 0.8905528 | 0.10404759 | 0.0052465612 | 1.502994e-4 | 2.709533e-6 |
| 12 | 0.8811884 | 0.11237259 | 0.0062362910 | 1.986080e-4 | 4.030109e-6 |
| 13 | 0.8719176 | 0.12052025 | 0.0073003763 | 2.558815e-4 | 5.772273e-6 |
| 14 | 0.8627396 | 0.12849312 | 0.0084364173 | 3.227535e-4 | 8.013131e-6 |
| 15 | 0.8536533 | 0.13629377 | 0.0096420591 | 3.998302e-4 | 1.083493e-5 |
| 16 | 0.8446580 | 0.14392469 | 0.0109149909 | 4.876911e-4 | 1.432479e-5 |
| 17 | 0.8357528 | 0.15138836 | 0.0122529452 | 5.868893e-4 | 1.857442e-5 |
| 18 | 0.8269369 | 0.15868724 | 0.0136536972 | 6.979526e-4 | 2.367987e-5 |
| 19 | 0.8182093 | 0.16582375 | 0.0151150645 | 8.213834e-4 | 2.974125e-5 |
